# The chromosomal genome sequence of the hump coral, *Porites cylindrica* Dana, 1846 (Scleractinia: Poritidae)

**DOI:** 10.12688/wellcomeopenres.27789.1

**Published:** 2026-09-14

**Authors:** Victor Bonito, Andrew H. Baird, Kate Quigley, Tom Bridge, Stefano Borghi, Leigh Peters, Camile Avelino, Sebastian Metz, Michael Sweet, Graeme Oatley, Elizabeth Sinclair, Eerik Aunin, Noah Gettle, Camilla Santos, Michael Paulini, Haoyu Niu, Victoria McKenna, Rebecca O’Brien, Wellcome Sanger Institute Tree of Life Management, Samples and Laboratory team, Wellcome Sanger Institute Scientific Operations: Sequencing Operations, Wellcome Sanger Institute Tree of Life Core Informatics team, EBI Aquatic Symbiosis Genomics Data Portal Team, Aquatic Symbiosis Genomics Project Leadership

**Affiliations:** 1 Reef Explorer Fiji, Votua Village, Fiji; 2 College of Science and Engineering, James Cook University, Townsville, Queensland, Australia; 3 Minderoo Foundation, Perth, Western Australia, Australia; 4 University of Western Australia, Perth, Western Australia, Australia; 5 Biodiversity and Geosciences Program, Queensland Museum Tropics, Queensland Museum, Townsville, Queensland, Australia; 6 Aquatic Research Facility, Nature-based Solutions Research Centre, University of Derby, Derby, England, UK; 7 Tree of Life, Wellcome Sanger Institute, Hinxton, England, UK

**Keywords:** Porites cylindrica, hump coral, genome sequence, chromosomal, Scleractinia

## Abstract

We present a genome assembly from a specimen of *Porites cylindrica* (hump coral; Cnidaria; Anthozoa; Scleractinia; Poritidae). The genome sequence has a total length of 589.27 megabases. Most of the assembly (99.25%) is scaffolded into 14 chromosomal pseudomolecules. The mitochondrial genome has also been assembled, with a length of 18.65 kilobases. Gene annotation of this assembly by Ensembl identified 25,774 protein-coding genes.

## Species taxonomy

Eukaryota; Opisthokonta; Metazoa; Eumetazoa; Cnidaria; Anthozoa; Hexacorallia; Scleractinia; Refertina; Poritidae; *Porites*; *Porites cylindrica* Dana, 1846 (NCBI:txid126659)

## Background

*Porites cylindrica* forms branching colonies, sometimes with an encrusting base, and can cover several square metres. The branches are smooth in appearance, with shallow corallites that give the colony a uniform appearance. Its colouration ranges from cream and yellow to blue, brown and green, although the species appears to be dominated by a single Symbiodiniaceae type, *Cladocopium* C15 ( Longley *et al.*, 2024).

*Porites cylindrica* was described by James Dana in 1846 from a single specimen collected in Fiji. It is present across the oceanic western Pacific, Australia, the South China Sea, Japan, Southeast Asia and the Indian Ocean ( Veron *et al.*, 2016). However, its range might be confounded by confusion with similar species such as *P. levis*, with which it coexists across at least part of this range. There are clear morphological differences between *P. levis* and *P. cylindrica*; for example, *P. levis* has thicker, longer branches. However, *P. levis* was synonymised with *P. cylindrica* by Veron and Pichon ( 1982).

*P. cylindrica* is a gonochoric species, with distinct male and female colonies releasing sperm and eggs separately. In Fiji, the species has been observed to spawn on the third night after the November full moon, approximately 2 hours after sunset. Vertical transmission of symbionts is also known ( Baird *et al.*, 2009).

*P. cylindrica* is assessed as Least Concern on the IUCN Red List ( Nuñez Lendo *et al.*, 2024).

**Figure 1.**
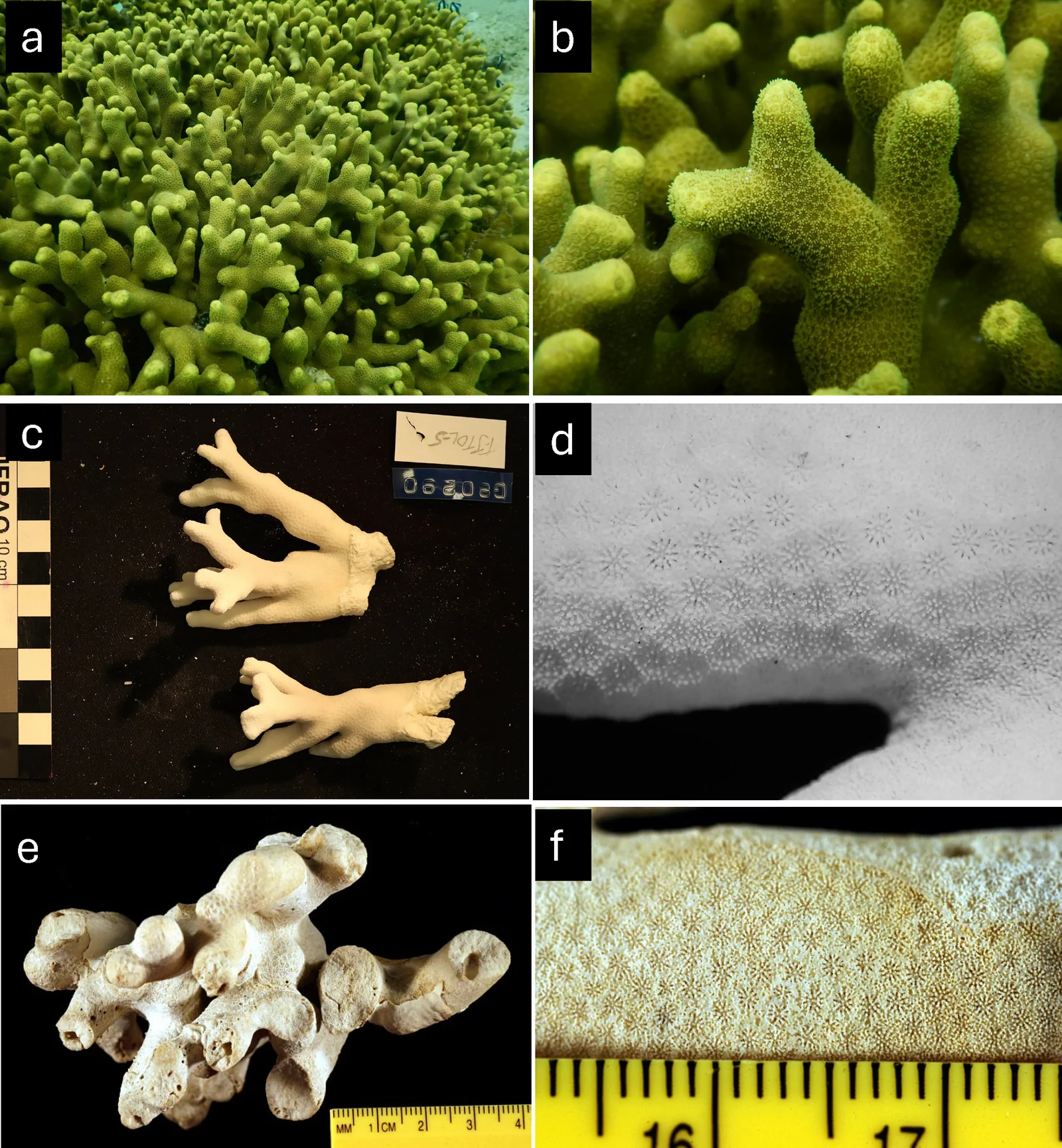
Colony, voucher specimen and syntype of *Porites cylindrica*. (a, b) Colony from which the voucher specimen was collected. (c, d) Voucher specimen G80290 (specimen ID UDUK0000339, ToLID jaPorCyli1). (e, f) Syntype USNM 708.

## Methods

### Sample acquisition

The voucher specimen was collected by hand with a screwdriver from 2m depth from a reef flat near Vatuolalai Village on the south-west coast of Fiji’s largest island, Viti Levu. The specimen was collected by Victor Bonito (Reef Explorer Fiji) and identified by Bonito and Andrew Baird (JCU). Tissue was removed from the specimen with a bone cutter and placed in DMSO and stored at 4°C. The remaining skeleton was bleached and dried and ultimately deposited at the Museum of Tropical Queensland in Townsville, Australia. Collected and exported under CITES permit 22FJ/NV200032.

The voucher specimen G80290 (specimen ID UDUK0000339, ToLID jaPorCyli1; Figure 1, panels c and d) was identified in the field as *Porites cylindrica* Dana 1846. The specimen has short branches that are cylindrical, crowded, and often furcate at the end. The corallites of the voucher are shallow giving a smooth appearance to the skeleton. The voucher is a good match for the syntype of *P. cylindrica* Dana 1846 (Figure 1, panels e and f).

### Nucleic acid extraction

Protocols for high molecular weight (HMW) DNA extraction developed at the Wellcome Sanger Institute (WSI) Tree of Life Core Laboratory are available on protocols.io ( Howard *et al.*, 2025). The jaPorCyli1 sample was weighed and triaged to determine the appropriate extraction protocol. Tissue was homogenised by cryogenic bead beating. HMW DNA was extracted using the Manual MagAttract v3 protocol. DNA was sheared into an average fragment size of 12–20 kb following the Megaruptor®3 for LI PacBio protocol. Sheared DNA was purified by automated SPRI (solid-phase reversible immobilisation). The concentration of the sheared and purified DNA was assessed using a NanoDrop spectrophotometer and Qubit Fluorometer using the Qubit dsDNA High Sensitivity Assay kit. Fragment size distribution was evaluated by running the sample on the FemtoPulse system. For this sample, the final post-shearing DNA had a Qubit concentration of 14.3 ng/μL and an average fragment size of 13.3 kb.

RNA was extracted from tissue of the jaPorCyli1 specimen in the Tree of Life Laboratory at the WSI using the RNA Extraction: Automated MagMax™ *mir*Vana protocol. The RNA concentration was assessed using a NanoDrop spectrophotometer and a Qubit Fluorometer using the Qubit RNA Broad-Range Assay kit. Analysis of the integrity of the RNA was done using the Agilent RNA 6000 Pico Kit and Eukaryotic Total RNA assay.

### PacBio HiFi library preparation and sequencing

Library preparation and sequencing were performed at the WSI Scientific Operations core. Libraries were prepared using the SMRTbell Prep Kit 3.0 (Pacific Biosciences, California, USA), following the manufacturer’s instructions. The kit includes reagents for end repair/A-tailing, adapter ligation, post-ligation SMRTbell bead clean-up, and nuclease treatment. Size selection and clean-up were performed using diluted AMPure PB beads (Pacific Biosciences). DNA concentration was quantified using a Qubit Fluorometer v4.0 (Thermo Fisher Scientific) and the Qubit 1X dsDNA HS assay kit. Final library fragment size was assessed with the Agilent Femto Pulse Automated Pulsed Field CE Instrument (Agilent Technologies) using the gDNA 55 kb BAC analysis kit.

The sample was sequenced using the Sequel IIe system (Pacific Biosciences, California, USA). The concentration of the library loaded onto the Sequel IIe was in the range 40–135 pM. SMRT Link software, a PacBio web-based workflow manager, was used to set up and monitor the run, and to perform primary and secondary analysis of the data upon completion.

### Hi-C

***Sample preparation and crosslinking***

The Hi-C sample was prepared from 20–50 mg of frozen tissue from the jaPorCyli1 sample using the Arima-HiC v2 kit (Arima Genomics). Following the manufacturer’s instructions, tissue was fixed and DNA crosslinked using TC buffer to a final formaldehyde concentration of 2%. The tissue was homogenised using the Diagnocine Power Masher-II. Crosslinked DNA was digested with a restriction enzyme master mix, biotinylated, and ligated. Clean-up was performed with SPRISelect beads before library preparation. DNA concentration was measured with the Qubit Fluorometer (Thermo Fisher Scientific) and Qubit HS Assay Kit. The biotinylation percentage was estimated using the Arima-HiC v2 QC beads.

***Hi-C library preparation and sequencing***

Biotinylated DNA constructs were fragmented using a Covaris E220 sonicator and size-selected to 400–600 bp using SPRISelect beads. DNA was enriched with Arima-HiC v2 kit Enrichment beads. End repair, A-tailing, and adapter ligation were carried out with the NEBNext Ultra II DNA Library Prep Kit (New England Biolabs), following a modified protocol where library preparation occurs while DNA remains bound to the Enrichment beads. Library amplification was performed using KAPA HiFi HotStart mix and a custom Unique Dual Index (UDI) barcode set (Integrated DNA Technologies). Depending on sample concentration and biotinylation percentage determined at the crosslinking stage, libraries were amplified with 10–16 PCR cycles. Post-PCR clean-up was performed with SPRISelect beads. Libraries were quantified using the AccuClear Ultra High Sensitivity dsDNA Standards Assay Kit (Biotium) and a FLUOstar Omega plate reader (BMG Labtech).

Prior to sequencing, libraries were normalised to 10 ng/μL. Normalised libraries were quantified again to create equimolar and/or weighted 2.8 nM pools. Pool concentrations were checked using the Agilent 4200 TapeStation (Agilent) with High Sensitivity D500 reagents before sequencing. Sequencing was performed using paired-end 150 bp reads on the Illumina NovaSeq X.

### RNA library preparation and sequencing

Libraries were prepared using the NEBNext® Ultra II Directional RNA Library Prep Kit for Illumina (New England Biolabs), following the manufacturer’s instructions. Poly(A) mRNA was isolated from 100 ng of total RNA using oligo(dT) beads, converted to cDNA, and uniquely indexed; 14 PCR cycles were performed. Libraries were prepared with an intended average fragment size of 100–300 bp. Libraries were quantified, normalised, and pooled equimolarly to a final concentration of 2.8 nM before dilution to 150 pM for loading. Sequencing was carried out on the Illumina NovaSeq X, generating paired-end reads.

### Genome assembly

Prior to assembly of the PacBio HiFi reads, a database of *k*-mer counts ( *k* = 31) was generated from the filtered reads using FastK. GenomeScope2 ( Ranallo-Benavidez *et al.*, 2020) was used to analyse the *k*-mer frequency distributions, providing estimates of genome size, heterozygosity, and repeat content.

The HiFi reads were assembled using Hifiasm ( Cheng *et al.*, 2021) with the --primary option. Haplotypic duplications were identified and removed using purge_dups ( Guan *et al.*, 2020). The Hi-C reads ( Rao *et al.* , 2014) were mapped to the primary contigs using bwa-mem2 ( Vasimuddin *et al.*, 2019), and the contigs were scaffolded in YaHS ( Zhou *et al.*, 2023) with the --break option for handling potential misassemblies. The scaffolded assemblies were evaluated using Gfastats ( Formenti *et al.* , 2022), BUSCO ( Manni *et al.*, 2021) and MerquryFK ( Rhie *et al.*, 2020).

The mitochondrial genome was assembled using MitoHiFi ( Uliano-Silva *et al.* , 2023). The MitoHiFi reference was the mitochondrial genome of *Porites harrisoni* (NC_037435.1).

### Assembly curation

The assembly was decontaminated using the Assembly Screen for Cobionts and Contaminants ( ASCC) pipeline. TreeVal was used to generate the flat files and maps for use in curation. Manual curation was conducted primarily in PretextView and HiGlass ( Kerpedjiev *et al.* , 2018). Scaffolds were visually inspected and corrected as described by Howe *et al*. (2021). Manual corrections included 24 breaks, 49 joins, and removal of 22 haplotypic duplications. This reduced the scaffold count by 16.1%, reduced the scaffold N50 by 28.9%, and reduced the total assembly length by 1.6%. The curation process is described at sanger-tol/curation-resources. PretextSnapshot was used to generate a Hi-C contact map of the final assembly.

### Assembly quality assessment

The Merqury.FK tool ( Rhie *et al.*, 2020) was run in a Singularity container ( Kurtzer *et al.*, 2017) to evaluate *k*-mer completeness and assembly quality for the primary and alternate haplotypes using the *k*-mer database ( *k* = 31) computed prior to genome assembly. The analysis outputs included assembly QV scores and completeness statistics.

The primary assembly was assessed using BlobToolKit ( Challis *et al.*, 2020). PacBio reads were mapped to the assembly to derive sequence coverage, and BUSCO ( Manni *et al.*, 2021) was used to assess gene-set completeness. DIAMOND BLASTp searches of extracted BUSCO proteins and DIAMOND BLASTx searches of assembly sequences against UniProt Reference Proteomes ( Buchfink *et al.*, 2021), followed by BLASTn searches of sequences without DIAMOND BLASTx hits against the NCBI nt database ( Altschul *et al.*, 1990), supported taxonomic assignment. Coverage, sequence composition, taxonomic assignment and BUSCO results were integrated into a BlobDir for interactive inspection and visualisation.

## Genome sequence report

### Sequence data

PacBio sequencing of the *Porites cylindrica* specimen generated 18.30 Gb (gigabases) from 2.00 million reads, which were used to assemble the genome. GenomeScope2.0 analysis using a *p* = 2 model gave a *k*-mer-based genome size estimate of 580.67 Mb, with a heterozygosity of 1.79% and repeat content of 38.76% (Figure 2). These estimates guided expectations for the assembly. Based on the estimated genome size, the sequencing data provided approximately 30× coverage. Hi-C sequencing produced 76.98 Gb from 254.90 million reads, which were used to scaffold the assembly. RNA sequencing data were also generated and are available in public sequence repositories. Table 1 summarises the specimen and sequencing details.

**Figure 2.**
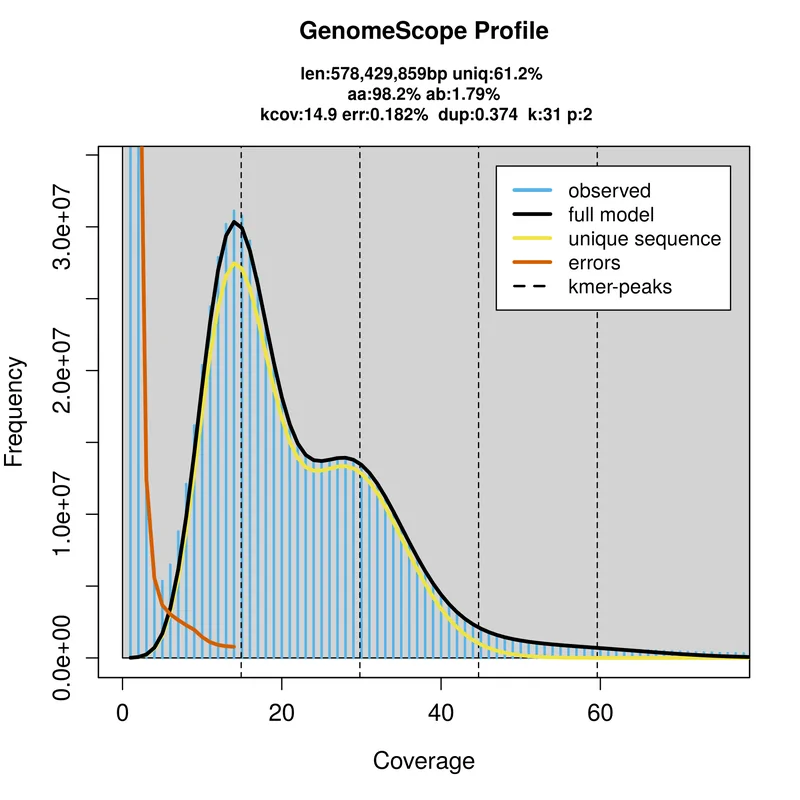
Frequency distribution of *k*-mers generated using GenomeScope2. The plot shows observed and modelled *k*-mer spectra, providing estimates of genome size, heterozygosity, and repeat content based on unassembled sequencing reads.

**Table 1.** Specimen and sequencing data for *Porites cylindrica* (BioProject PRJEB74960)

**Platform**	**PacBio HiFi**	**Hi-C**	**RNA-seq**
**ToLID**	jaPorCyli1	jaPorCyli1	jaPorCyli1
**Specimen ID**	UDUK0000339	UDUK0000339	UDUK0000339
**BioSample (source individual)**	SAMEA110640074	SAMEA110640074	SAMEA110640074
**BioSample (tissue)**	SAMEA110640157	SAMEA110640161	SAMEA110640159
**Instrument**	Sequel IIe	Illumina NovaSeq X	Illumina NovaSeq X
**Run accessions**	ERR12921310	ERR12945464	ERR13669975
**Read count total**	2.00 million reads	254.90 million read pairs	10.02 million read pairs
**Base count total**	18.30 Gb	76.98 Gb	3.03 Gb

### Assembly statistics

The primary haplotype was assembled, and contigs corresponding to an alternate haplotype were also deposited in INSDC databases. The final assembly has a total length of 589.27 Mb in 108 scaffolds, with 182 gaps, and a scaffold N50 of 37.87 Mb The primary assembly was generated and an alternate haplotype assembly was also deposited at scaffold level in INSDC databases. The primary assembly is proposed as the reference assembly for this species. It has a total length of 589.27 Mb in 108 scaffolds (excluding organelle sequences), with 182 gaps, and a scaffold N50 of 37.87 Mb (Table 2).

**Table 2.** Genome assembly data for *Porites cylindrica*

**Genome assembly**	**Primary assembly**	**Alternate haplotype assembly**
**Assembly name**	jaPorCyli1.1	jaPorCyli1.1 alternate haplotype
**Assembly accession**	GCA_964035525.1	GCA_964035495.1
**Assembly level**	chromosome	scaffold
**Span (Mb)**	589.27	596.24
**Number of chromosomes**	14	-
**Number of contigs**	290	2,664
**Contig N50 (Mb)**	5.28	0.94
**Number of scaffolds (excluding organelle sequences)**	108	2,663
**Scaffold N50 (Mb)**	37.87	0.94
**Scaffold auN (Mb)**	46.1	-
**Longest scaffold length (Mb)**	68.68	6.47
**Organelles**	Mitochondrial genome: 18.65 kb	-

Most of the assembly sequence (99.25%) was assigned to 14 chromosome-level scaffolds. These chromosome-level scaffolds, confirmed by Hi-C data, are named according to size (Figure 3; Table 3).

The mitochondrial genome was also assembled (length 18.65 kb, OZ037789.1). This sequence is included as a contig in the multifasta file of the genome submission and as a standalone record.

**Figure 3.**
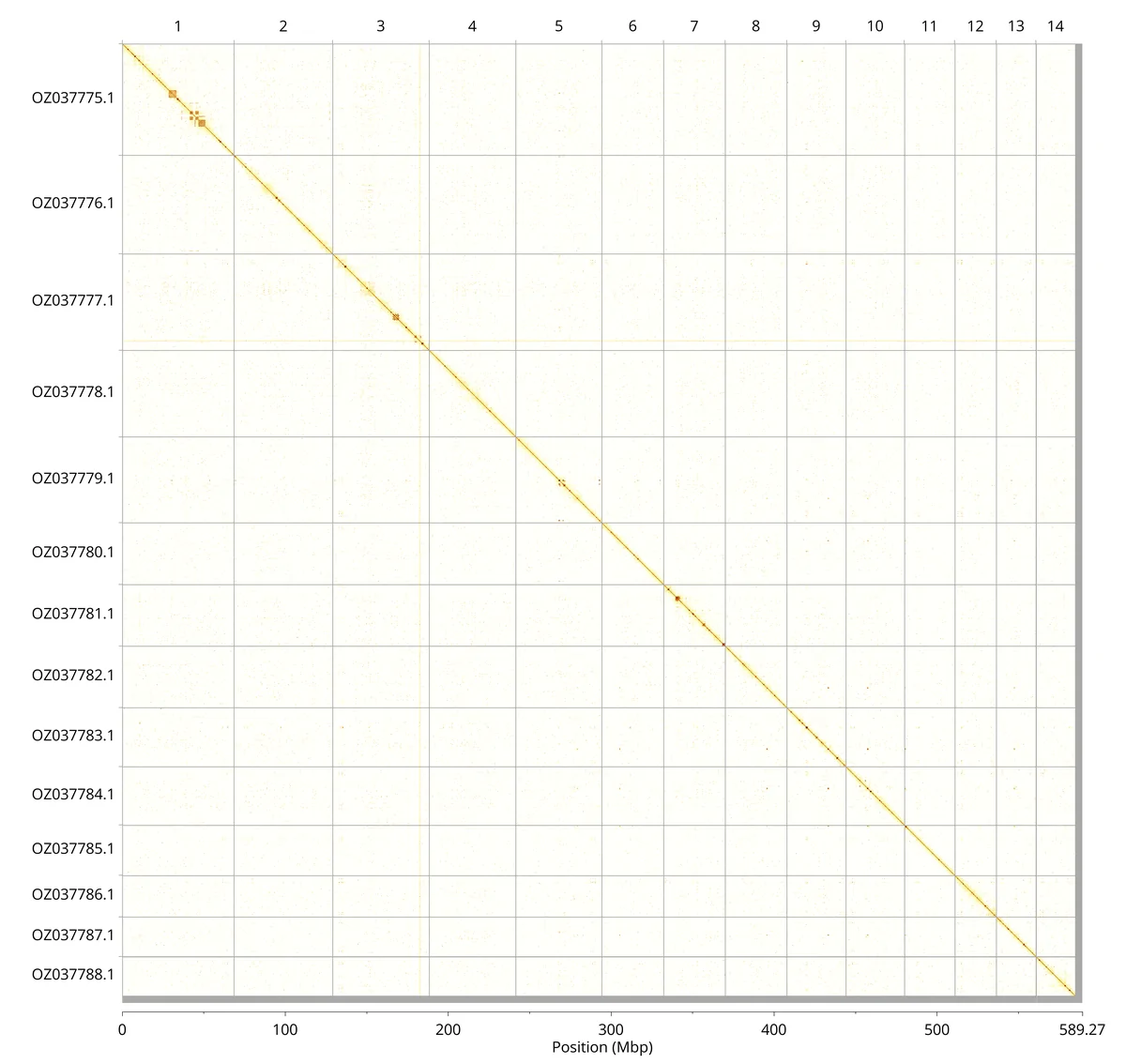
Hi-C contact map of the *Porites cylindrica* genome assembly. Assembled chromosomes are shown in order of size and labelled along the axes, with a megabase scale shown below. The plot was generated using PretextSnapshot.

**Table 3.** Chromosomal pseudomolecules in the primary genome assembly of *Porites cylindrica* jaPorCyli1

**INSDC accession**	**Molecule**	**Length (Mb)**	**GC%**
OZ037775.1	1	68.68	39
OZ037776.1	2	60.48	39
OZ037777.1	3	59.20	39.50
OZ037778.1	4	53.20	39
OZ037779.1	5	52.76	39
OZ037780.1	6	37.87	39
OZ037781.1	7	37.84	39
OZ037782.1	8	37.81	39
OZ037783.1	9	36.27	39.50
OZ037784.1	10	35.86	39
OZ037785.1	11	30.92	39
OZ037786.1	12	25.48	39
OZ037787.1	13	24.41	39
OZ037788.1	14	24.04	39

### Assembly quality metrics

The combined primary and alternate assemblies achieve an estimated QV of 60.1. The *k*-mer completeness is 70.81% for the primary assembly, 68.14% for the alternate haplotype, and 99.23% for the combined assemblies (Figure 4).

**Figure 4.**
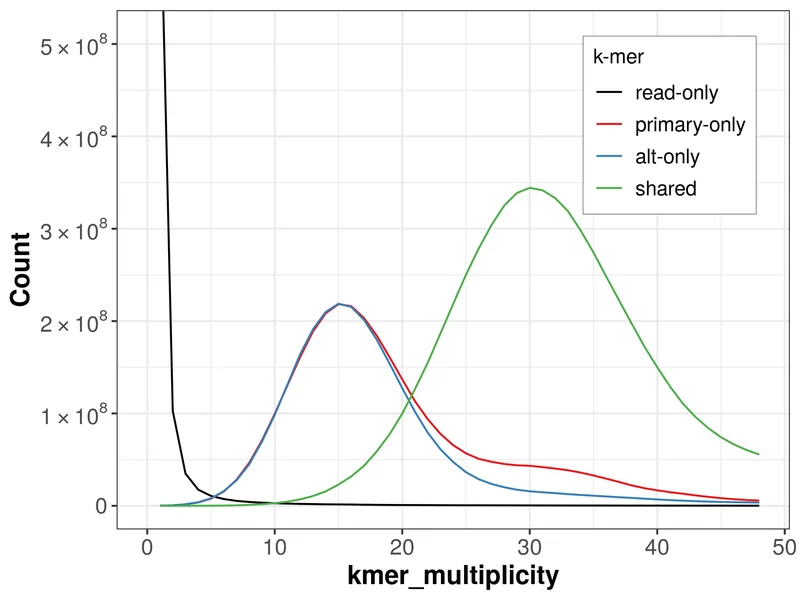
Evaluation of *k*-mer completeness using MerquryFK. This plot illustrates the recovery of *k*‐mers from the original read data in the final assemblies. The horizontal axis represents *k*‐mer multiplicity, and the vertical axis shows the number of *k*‐mers. The black curve represents *k*‐mers that appear in the reads but are not assembled. The green curve corresponds to *k*‐mers shared by both haplotypes, and the red and blue curves show *k*‐mers found only in one of the haplotypes.

BUSCO v.5.5.0 analysis using the metazoa_odb10 reference set ( *n* = 954) identified 96.0% of the expected gene set (single = 95.0%, duplicated = 1.0%). The snail plot in Figure 5 summarises the scaffold length distribution and other assembly statistics for the primary assembly. The blob plot in Figure 6 shows the distribution of scaffolds by GC proportion and coverage.

**Figure 5.**
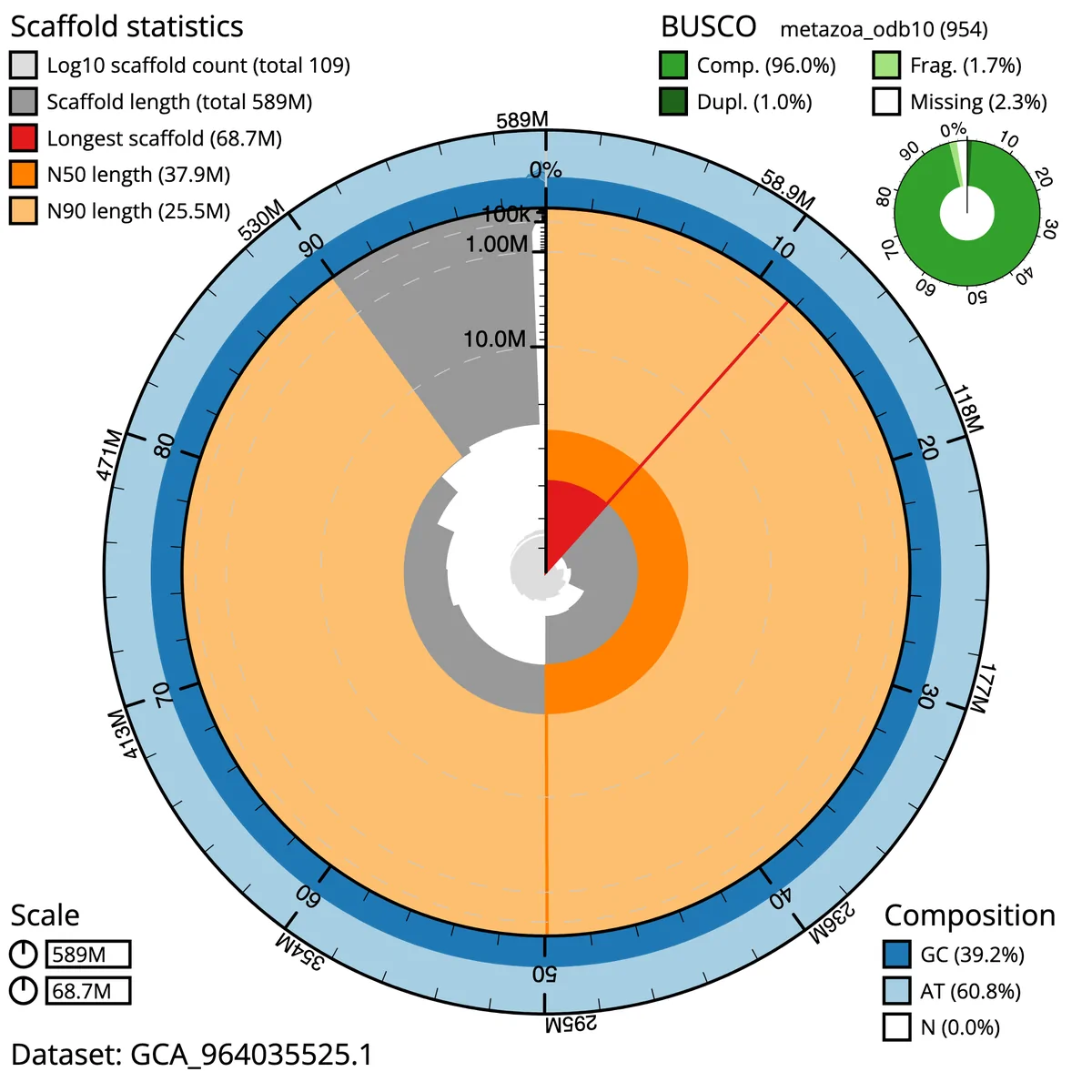
Assembly metrics for jaPorCyli1.1. The BlobToolKit snail plot provides an overview of assembly metrics and BUSCO gene completeness. The circumference represents the length of the whole genome sequence, and the main plot is divided into 1,000 bins around the circumference. The outermost blue tracks display the distribution of GC, AT, and N percentages across the bins. Scaffolds are arranged clockwise from longest to shortest and are depicted in dark grey. The longest scaffold is indicated by the red arc, and the deeper orange and pale orange arcs represent the N50 and N90 lengths. A light grey spiral at the centre shows the cumulative scaffold count on a logarithmic scale. A summary of complete, fragmented, duplicated, and missing BUSCO genes in the metazoa_odb10 set is presented at the top right. An interactive version of this figure can be accessed on the BlobToolKit viewer.

**Figure 6.**
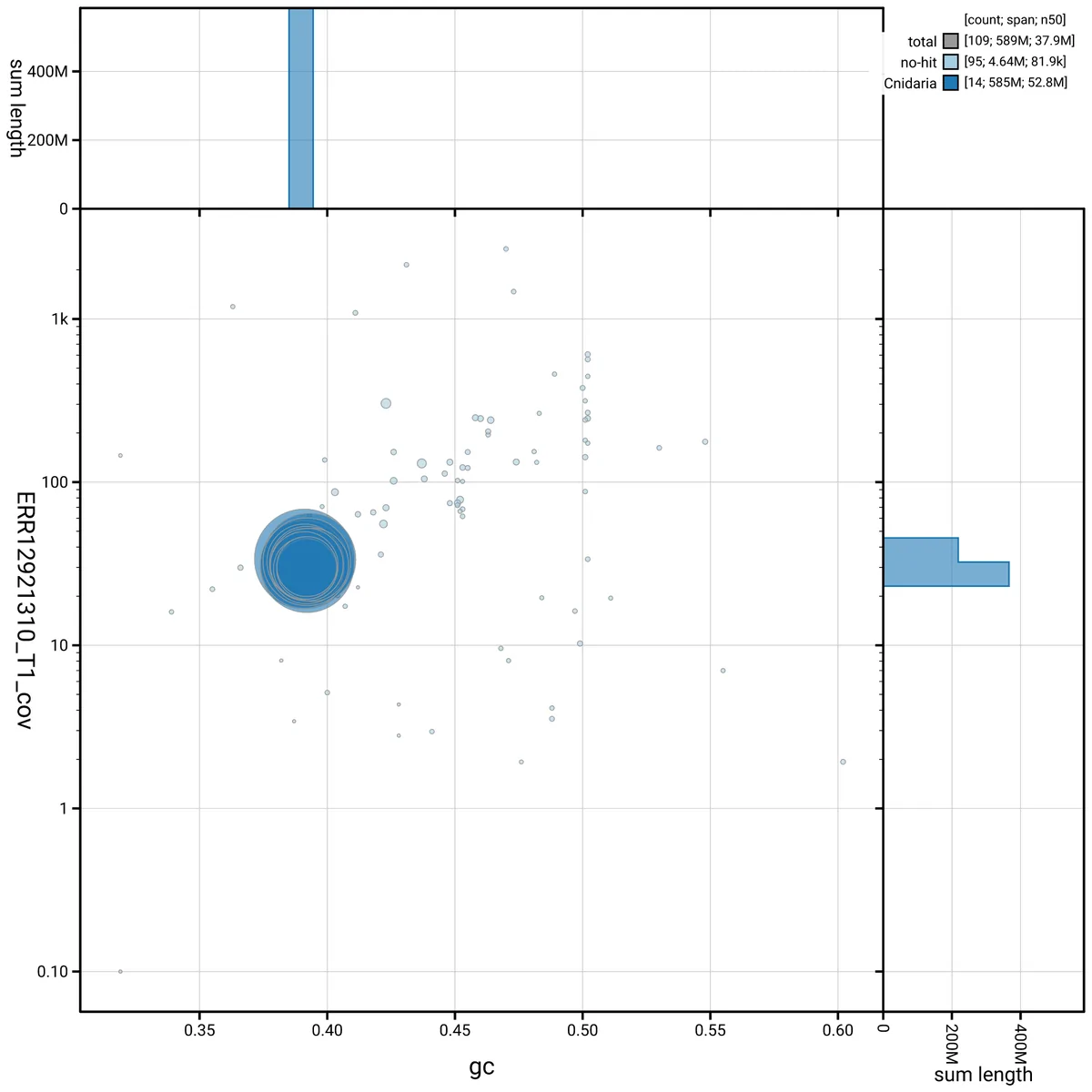
BlobToolKit blob plot for jaPorCyli1.1. The plot shows base coverage (vertical axis) and GC content (horizontal axis). The circles represent scaffolds, with the size proportional to scaffold length and the colour representing phylum membership. The histograms along the axes display the total length of sequences distributed across different levels of coverage and GC content. An interactive version of this figure is available on the BlobToolKit viewer.

Table 4 lists the assembly metric benchmarks adapted from Rhie *et al.*. (2021) and the Earth BioGenome Project (EBP) Report on Assembly Standards January 2026. The EBP metric, calculated for primary assembly, is **6.C.Q60**, meeting the recommended 6.C.Q40 reference standard.

**Table 4.** Earth Biogenome Project summary metrics for the *Porites cylindrica* assembly

**Measure**	**Value**	***Benchmark***
EBP summary (primary)	6.C.Q60	*6.C.Q40*
Contig N50 length	5.28 Mb	*> 1 Mb*
Scaffold N50 length	37.87 Mb	*= chromosome N50*
Consensus quality (QV)	Primary: 60.6; alternate: 59.8; combined: 60.1	*≥ 40*
*k*-mer completeness	Primary: 70.81%; alternate: 68.14%; combined: 99.23%	*> 90%*
BUSCO	C:96.0% [S:95.0%, D:1.0%], F:1.7%, M:2.3%, n:954	*S > 90%; D < 5%*
Percentage of assembly assigned to chromosomes	99.25%	*≥ 90%*

**Notes:** The EBP summary uses log10(Contig N50); chromosome-level (C) or log10(Scaffold N50); Q (Merqury QV). BUSCO: C=complete; S=single-copy; D=duplicated; F=fragmented; M=missing; n=orthologues

### Genome annotation report

The *Porites cylindrica* genome assembly (GCA_964035525.1) was annotated by Ensembl at the European Bioinformatics Institute (EBI). This annotation includes 52,956 transcribed mRNAs from 25,774 protein-coding and 13,185 non-coding genes. The average transcript length is 10,480.53 bp, with an average of 1.36 coding transcripts per gene and 6.03 exons per transcript. Further details of this annotation are available from the Ensembl annotation page.

The genome was also annotated by Metz *et al.*. (2026) using BRAKER3 v3.0.8 ( Gabriel *et al.*, 2024). The annotation comprises 34,276 coding sequences, with an average gene length of 1,402 bp, 1.13 coding transcripts per gene, and 6.07 exons per transcript. Annotation completeness was assessed using BUSCO v5.8.2 against the cnidaria_odb12 lineage dataset, yielding 97.4% complete (85.0% single-copy, 12.4% duplicated), 1.0% fragmented, and 1.6% missing BUSCOs. Annotation files are available from the GitHub repository.

### Data availability

European Nucleotide Archive: Porites cylindrica. Accession number PRJEB74960; https://identifiers.org/ena.embl/PRJEB74960. The genome sequence is released openly for reuse. The *Porites cylindrica* genome sequencing initiative is part of the Aquatic Symbiosis Genomics Project ( PRJEB43743) and the Sanger Institute Tree of Life Programme ( PRJEB43745). All raw sequence data and the assembly have been deposited in INSDC databases. Raw data and assembly accession identifiers are reported in Tables 1 and 2.

Production code used in genome assembly at the WSI Tree of Life is available at https://github.com/sanger-tol. Table 5 lists software versions used in this study.

**Table 5.** Software versions and sources used for *Porites cylindrica*

**Software**	**Version**	**Source**
BLAST	2.14.0	https://ftp.ncbi.nlm.nih.gov/blast/executables/blast+/
blobtk	0.5.1	https://github.com/genomehubs/blobtk
BlobToolKit	4.3.9	https://github.com/genomehubs/blobtoolkit
BUSCO	5.5.0; 6.1.0	https://gitlab.com/ezlab/busco
bwa-mem2	2.2.1	https://github.com/bwa-mem2/bwa-mem2
DIAMOND	2.1.8	https://github.com/bbuchfink/diamond
fasta_windows	0.2.4	https://github.com/tolkit/fasta_windows
FastK	1.1	https://github.com/thegenemyers/FASTK
GenomeScope2.0	2.0.1	https://github.com/tbenavi1/genomescope2.0
Gfastats	1.3.6	https://github.com/vgl-hub/gfastats
Hifiasm	0.19.8-r603	https://github.com/chhylp123/hifiasm
HiGlass	1.13.4	https://github.com/higlass/higlass
MerquryFK	1.1.0-c1	https://github.com/thegenemyers/MERQURY.FK
Minimap2	2.24-r1122	https://github.com/lh3/minimap2
MitoHiFi	3.2.2	https://github.com/marcelauliano/MitoHiFi
MultiQC	1.14; 1.17 and 1.18	https://github.com/MultiQC/MultiQC
Nextflow	23.10.0; 25.10.0; 25.10.4	https://github.com/nextflow-io/nextflow
PretextSnapshot	0.0.6	https://github.com/sanger-tol/PretextSnapshot
PretextView	1.0.3	https://github.com/sanger-tol/PretextView
purge_dups	1.2.3	https://github.com/dfguan/purge_dups
samtools	1.16.1; 1.17; 1.18; 1.19.2; 1.6	https://github.com/samtools/samtools
sanger-tol/ascc	0.1.0	https://github.com/sanger-tol/ascc
sanger-tol/blobtoolkit	0.6.0	https://github.com/sanger-tol/blobtoolkit
sanger-tol/curationpretext	1.4.2	https://github.com/sanger-tol/curationpretext
Seqtk	1.4-r122	https://github.com/lh3/seqtk
Singularity	3.9.0	https://github.com/sylabs/singularity
TreeVal	1.0.2; 1.1.0	https://github.com/sanger-tol/treeval
YaHS	1.1a.2	https://github.com/c-zhou/yahs

### Author information

Contributors are listed at the following links:

- Members of the Wellcome Sanger Institute Tree of Life Management, Samples and Laboratory team
- Members of Wellcome Sanger Institute Scientific Operations – Sequencing Operations
- Members of the Wellcome Sanger Institute Tree of Life Core Informatics team
- Members of the EBI Aquatic Symbiosis Genomics Data Portal Team
- The Aquatic Symbiosis Genomics Project leadership

### Wellcome Sanger Institute – Legal and Governance

The materials that have contributed to this genome note have been supplied by a Tree of Life collaborator. The Wellcome Sanger Institute employs a process whereby due diligence is carried out proportionate to the nature of the materials themselves, and the circumstances under which they have been/are to be collected and provided for use. The purpose of this is to address and mitigate any potential legal and/or ethical implications of receipt and use of the materials as part of the research project, and to ensure that in doing so we align with best practice wherever possible.

The overarching areas of consideration are:

- Ethical review of provenance and sourcing of the material
- Legality of collection, transfer and use (national and international)

Each transfer of samples is undertaken according to a Research Collaboration Agreement or Material Transfer Agreement entered into by the Tree of Life collaborator, Genome Research Limited (operating as the Wellcome Sanger Institute) and in some circumstances other Tree of Life collaborators.
