# Supplementary material for: The chromosomal genome sequence of the hump coral, Porites cylindrica Dana, 1846 (Scleractinia: Poritidae)
Source: Wellcome Open Res. 2026 Sep 14;11:648. [Version 1] doi: 10.12688/wellcomeopenres.27789.1 (PMC13710864; doi:10.12688/wellcomeopenres.27789.1)
Supplement: Peer review history

# Reviewer report: Reviewer response for version 1

**DOI:** 10.21956/wellcomeopenres.30582.r171108

**Published:** 2026-11-02

**Authors:** Adrián Jaimes-Becerra

**Affiliations:** 1 Tohoku University, Sendai, Japan

This manuscript presents a valuable chromosome-scale genome assembly of *Porites cylindrica*, supported by PacBio HiFi and Hi-C sequencing, and provides a useful resource for coral genomics.

 I only have two comments:

 - The Ensembl annotation contains 25,774 protein-coding genes, whereas BRAKER3 reports 34,276 coding sequences. Please briefly discuss the potential reasons for the difference. The reported length statistics also require clarification, particularly whether they refer to genomic gene spans, spliced transcripts, or coding sequences.

 - You could briefly summarize the number, total length, and taxonomic assignments, where available, of sequences removed during decontamination.

Are the datasets clearly presented in a usable and accessible format, and the assembly and annotation available in an appropriate subject-specific repository?

Yes

Are sufficient details of the sequencing and extraction, software used, and materials provided to allow replication by others?

Yes

Are the rationale for sequencing the genome and the species significance clearly described?

Yes

Are the protocols appropriate and is the work technically sound?

Yes

Reviewer Expertise:

Evolutionary biology, Cnidaria genomics, venom evolution.

I confirm that I have read this submission and believe that I have an appropriate level of expertise to confirm that it is of an acceptable scientific standard.

# Reviewer report: Reviewer response for version 1

**DOI:** 10.21956/wellcomeopenres.30582.r173314

**Published:** 2026-10-26

**Authors:** Bingpeng Xing

**Affiliations:** 1 Third Institute of Oceanography Ministry of Natural Resources, Xiamen, Fujian, China

This genome note presents a high-quality chromosomal-level genome assembly for the hump coral, *Porites cylindrica*. The total assembly length spans 589.27 megabases, with 99.25% of the sequence successfully scaffolded into 14 chromosomal pseudomolecules. Utilizing advanced platforms such as PacBio HiFi and Hi-C data, the work achieves remarkable technical standards, including an estimated QV of 60.1 and a BUSCO gene completeness score of 96.0%. Furthermore, all experimental protocols, software versions, and datasets are comprehensively documented and made openly accessible in public repositories like the European Nucleotide Archive, ensuring complete reproducibility for future research.

Are the datasets clearly presented in a usable and accessible format, and the assembly and annotation available in an appropriate subject-specific repository?

Yes

Are sufficient details of the sequencing and extraction, software used, and materials provided to allow replication by others?

Yes

Are the rationale for sequencing the genome and the species significance clearly described?

Yes

Are the protocols appropriate and is the work technically sound?

Yes

Reviewer Expertise:

marine biology，genome

I confirm that I have read this submission and believe that I have an appropriate level of expertise to confirm that it is of an acceptable scientific standard.

# Reviewer report: Reviewer response for version 1

**Published:** 2026-09-14

**Authors:** Automated Benchmarking

**Affiliations:** 1 Wellcome Sanger Institute, Hinxton, UK

As this article is a Genome Note, Automated Benchmarking has been used to determine whether the reported assembly passes three defined checks established by the Earth BioGenome Project: the raw and assembled data are publicly available, the Earth BioGenome Project three-part metric is met, and the BUSCO score passes the stated threshold. For Porites cylindrica (jaPorCyli1), all three checks passed. Data available: pass. ENA study PRJEB74960, raw-read accessions ERR12921310 and ERR12945464, and assembly accessions GCA_964035525.1 and GCA_964035495.1 are public. Earth BioGenome Project three-part metric: pass. The primary assembly is reported as 6.C.Q60, meeting the 6.C.Q40 reference standard. BUSCO: pass. The primary assembly, which is proposed as the reference assembly for this species, has 96.0% complete BUSCOs (95.0% single-copy and 1.0% duplicated). BUSCO software version: 5.5.0; lineage dataset: metazoa_odb10. It meets the target of greater than 90% complete and single-copy BUSCOs. This decision uses values reported in the Genome Note and public accession records.

Reviewer Expertise:

NA

I confirm that I have read this submission and believe that I have an appropriate level of expertise to confirm that it is of an acceptable scientific standard.
